# Ectopic Cushing's Syndrome Unveiling a Metastatic Parotid Carcinoma

**DOI:** 10.1155/2019/3196283

**Published:** 2019-10-15

**Authors:** Sofia Castro Oliveira, João Sérgio Neves, Pedro Souteiro, Sandra Belo, Ana Isabel Oliveira, Helena Moreira, Paulo Mergulhão Gomes, Lígia Coelho, Cristina Sarmento, Elsa Fonseca, Celestino Neves, Paula Freitas, Davide Carvalho

**Affiliations:** ^1^Department of Endocrinology, Diabetes and Metabolism, Centro Hospitalar Universitário de São João (CHSJ), Porto, Portugal; ^2^Faculty of Medicine of Universidade do Porto (FMUP), Porto, Portugal; ^3^Instituto de Investigação e Inovação em Saúde, Universidade do Porto (i3S), Porto, Portugal; ^4^Unidade Polivalente de Cuidados Intermédios Geral (UPCIG), Centro Hospitalar Universitário de São João (CHSJ), Porto, Portugal; ^5^Department of Maxillofacial Surgery, Centro Hospitalar Universitário de São João (CHSJ), Porto, Portugal; ^6^Department of Medical Oncology, Centro Hospitalar Universitário de São João (CHSJ), Porto, Portugal; ^7^Department of Pathology, Centro Hospitalar Universitário de São João (CHSJ), Porto, Portugal; ^8^Instituto de Patologia e Imunologia Molecular, Universidade do Porto (IPATIMUP), Porto, Portugal

## Abstract

**Introduction:**

Adrenocorticotropic hormone (ACTH) ectopic production is a rare cause of Cushing syndrome (CS). The most commonly associated tumours are small-cell lung carcinoma along with bronchial and thymic carcinoids. To date, only 5 cases have been published in the literature featuring ectopic ACTH secretion from metastatic acinic cell carcinoma (ACC) of the parotid gland. We hereby describe a very uncommon case of ectopic CS (ECS) unveiling a metastatic parotid ACC.

**Case Presentation:**

A 46-year-old man with hypertension and dyslipidemia diagnosed 4-months before, as well as new-onset diabetes mellitus unveiled 1-month earlier, was referred to emergency department for hypokalemia. Hormonal study and dynamic biochemical tests performed indicated ECS. Imaging and cytological findings pointed toward a likely primary right parotid malignancy with liver metastases. Somatostatin receptor scintigraphy has shown an increased uptake in the parotid gland and mild expression in liver metastasis. The patient underwent right parotidectomy, and histopathologic examination confirmed ACC. Meanwhile, hypercortisolism was managed with metyrapone, ketoconazole, and lanreotide. Despite chemotherapy onset, a rapid disease progression and clinical course deterioration was observed.

**Conclusion:**

The present report highlights a rare ECS, exposing a metastatic parotid ACC, with an aggressive and challenging clinical course, representing the first case whose diagnosis of ECS came prior to ACC.

## 1. Introduction

Adrenocorticotropic hormone (ACTH) ectopic production is a rare cause of Cushing syndrome (CS), accounting for only 6% of the cases in a recent multicenter study [1, 2]. The most commonly associated tumours are small-cell lung carcinoma (SCLC) along with bronchial and thymic carcinoids [3, 4]. To date, to the best of our knowledge, only 5 cases have been published in the literature featuring ectopic ACTH secretion from metastatic acinic cell carcinoma (ACC) of the parotid gland. In fact, primary ACC springing up from salivary glands represents a very uncommon, typically low-grade malignant tumour, accounting for 1%–6% of the salivary neoplasms and 15% of all parotid gland malignancies [5–7]. ACC affects mainly mid-age females, metastasize in about 10% of the cases and 35% tends to recur, with a 5-year disease-associated mortality usually less than 10% [5, 7].

We hereby report a rare case of an ectopic CS (ECS) unveiling a metastatic parotid ACC, with an aggressive and defiant clinical course.

## 2. Case Presentation

We describe a case of a 46-year-old man with hypertension and dyslipidemia diagnosed 4-months before, as well as new-onset diabetes mellitus (DM) unveiled 1 month earlier (on oral anti-diabetic drugs), referred to emergency department for hypokalemia of 2.5 mEq/L [reference value (RV): 3.5–5.1]. He had paresthesias, weakness, anorexia, and asthenia associated with a marked weight loss (about 10 kg) for over a month, along with a suspicion of endogenous hypercortisolism [morning ACTH 146.0 ng/L (RV: 9.0–52.0) and cortisol 44.5 *μ*g/dL (RV: 5.0–25.0)] on ambulatory analysis. The patient also presented mild peripheral oedema, in the absence of striae or typical central obesity. He was then admitted to the Endocrinology department for further investigation.

Hormonal study revealed a midnight serum cortisol level of 36.2 ug/dL (RV: <7.5), midnight salivary cortisol 4.2 ug/dL (RV: <0.3) and 24 h-urinary free cortisol (UFC) 6210.0 ug/24 h (RV: 36.0–137.0). The result of 1 mg overnight dexamethasone suppression test was 42.6****ug/dL (RV: <1.8) (Table 1). The remaining biochemical assessment indicated serum potassium level of 3.0 mEq/L (RV: 3.5–5.1), with no other relevant changes. We subsequently performed a low-dose dexamethasone suppression test, which was compatible with CS, and a further high-dose dexamethasone suppression test showed no suppression of cortisol levels (Table 2).

**Figure 1:**
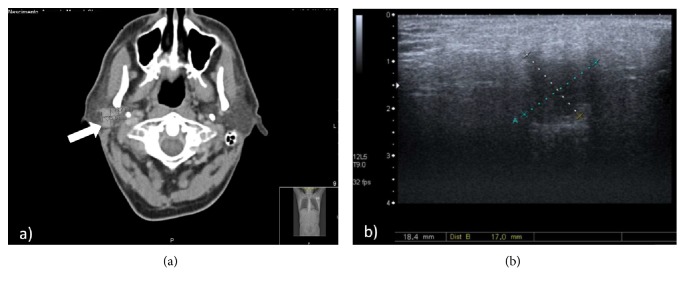
Cervico-thoraco-abdominal CT scan exposed a 16 × 1 × 17 mm nodular image on the right parotid gland (a); Parotid ultrasound documented a non specific 18 mm hypoechogenic nodule in the right parotid gland, with no signs of vascular invasion and no cervical adenomegalies (b).

Initial abdominal ultrasound found multiple nodular lesions in right hepatic lobe, the largest with 65 mm diameter, suggestive of secondary lesions by neoplastic process. Cervico-thoraco-abdominal computed tomography (CT) scan exposed a 16 × 1 × 17 mm nodular image on the right parotid gland (Figure 1(a)). Upper endoscopy and colonoscopy showed no significant lesions. Parotid ultrasound, subsequently performed, documented a non specific 18 mm hypoechogenic nodule in the right parotid gland, with no signs of vascular invasion or cervical adenomegalies (Figure 1(b)).

**Figure 2:**
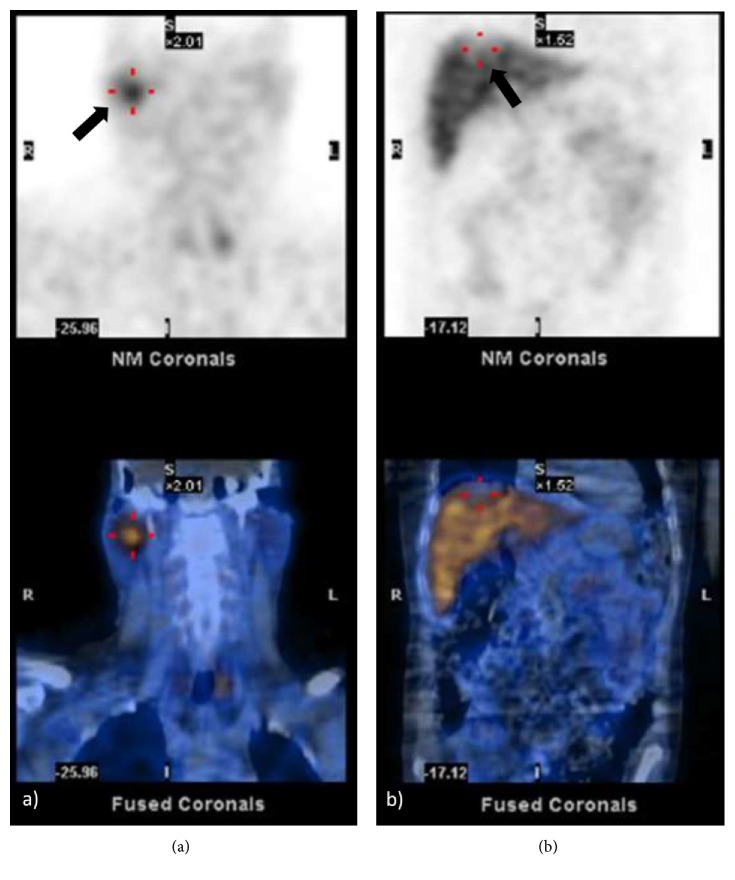
Octreoscan revealed increased uptake in the parotid gland (a) and mild expression in liver metastases (b).

Notably, parotid fine-needle aspiration biopsy revealed carcinoma (primary or metastatic), raising the suspicion of an ACC. A deeper investigation was carried out, with cervical magnetic resonance imaging (MRI) revealing a 17 × 17 × 20 mm spiculated nodule in the superficial lobe of the right parotid, immediately posterior to the mandible angle, with a marked contrast-uptake; in addition, a 9 mm well-circumscribed nodule in the deeper planes of the left submandibular gland was reported, without malignancy features; no other cervical lesions were observed. Liver biopsy confirmed malignancy, with overlapping morphological features of the parotid cytology. Somatostatin receptor scintigraphy (Octreoscan) demonstrated an increased uptake in the parotid gland (Figure 2(a)), whereas liver metastases exhibited mild expression (Figure 2(b)). Meanwhile, ^68^Gallium (^68^Ga)-Positron emission tomography (PET) showed no uptake.

The patient underwent right parotidectomy, with concomitant right supraomohyoid neck dissection and left submaxillectomy. The postoperative period was complicated by local hematoma with airway compromise, requiring surgical reintervention which, subsequently, led to an altered state of consciousness. In face of a normal brain CT, a psychosis associated with hypercortisolism was considered. In addition, he also presented infection of the surgical site, solved after antibiotherapy course.

**Figure 3:**
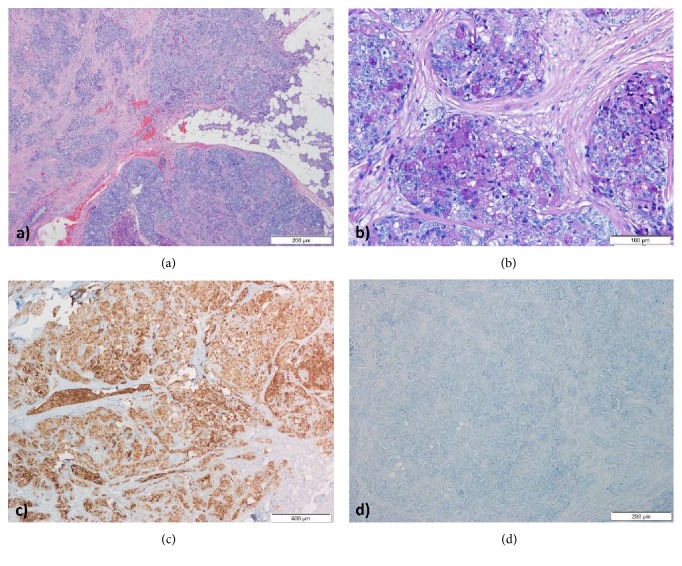
Histology–ACC: solid pattern and necrosis (H&E staining) (a); typical acinar cells with diastase-resistant PAS-positive granules (PAS-D staining) (b). The tumour cells showed strong immunoreactivity for DOG1 (c), being negative for ACTH immunostaining (d). ACC: acinic cell carcinoma; H&E: hematoxylin and eosin; PAS: periodic acid–schiff; PAS-D: PAS–diastase; DOG1: discovered on GIST-1; ACTH: adrenocorticotropic hormone.

Pathologic examination showed an ACC: solid patterned neoplasia, without glandular features or evidence of mucin secretion, composed by cells with extensive acinic differentiation demonstrated by the presence of diastase-resistant PAS-positive zymogen granules that were immunoreactive for cytokeratins 8/18, DOG1, beta-catenin, and cyclin D1; Ki67 index 70%. There was no evidence of neuroendocrine differentiation (the neoplastic cells were immunonegative for chromogranin A and synaptophysin.). Immunoexpression of ACTH was not detected (Figure 3). Pathologic TNM staging: pT3N0R1.

Imaging reevaluation indicated disease progression with, additionally, multiple bone metastases in vertebral column. Thereafter, chemotherapy was started with a weekly paclitaxel-carboplatin regimen and hypercortisolism was managed with metyrapone (3 g/day), ketoconazole (400 mg/day) and lanreotide (120 mg/month). Further, high insulin doses were required to maintain an acceptable glycemic control. Posterior to case discussion at multidisciplinary team meeting, the patient was proposed to radiotherapy of symptomatic spinal metastases. Afterwards, a rapid disease progression was depicted by cervico-thoraco-abdominal CT with hepatic, bone, adrenal, and ganglionar metastases. At that time, it was decided to switch chemotherapy regimen to oral vinorelbine in monotherapy. However, in face of the clinical worsening, that treatment was withdrawn and the patient was referred to palliative care. As his condition progressively deteriorated, he eventually passed away, 15-months after initial diagnosis.

## 3. Discussion

The authors describe an ECS emerging as a paraneoplastic feature, exposing a metastatic parotid ACC, with a challenging and fierce outcome.

Endogenous CS is a rare endocrine entity, with evidence pointing toward an annual incidence of 0.2–5 cases per million people, being about 80% of the cases ACTH-dependent forms [1, 8]. Among these, pituitary corticotroph adenoma (Cushing's disease) is the most common, with extrapituitary (ectopic) tumours standing for up to 20% [9]. Recent data from the large ERCUSYN cohort, points out that only 6% of total CS cases was caused by ectopic ACTH secretion [2]. SCLC along with pancreatic, thymic and bronchial neuroendocrine tumours (NETs) accounts for the majority of ECS cases. Other less frequently associated tumours comprise medullary carcinoma of thyroid, pheochromocytoma, gastroenteropancreatic and genitourinary tumours [3, 4, 10]. Noteworthy, parotid origin is extremely rare, with only 5 cases drawn in the literature so far (summarized on Table 3) [10–14].

Salivary gland carcinomas constitute rare malignant tumours comprising a diverse set of histopathology features [7]. These entities can be classified as either high-grade or low-grade tumours such as salivary duct carcinomas and conventional ACC, respectively [7]. Among the latter, mostly springs up from the major glands, with about 80% arising in the parotid gland [15]. In effect, parotid ACC is typically a slow-growing tumour, albeit owing the potential to recur locally or metastasize, with the lung, bone and liver typifying the commonest locations affected [15]. The 5-year disease-associated mortality ranges from 1.3% to 26% in the available literature [15].

Whilst parotid ACC etiology is yet to be fully disclosed, ionizing radiation and familiar predisposition represent well-established risk factors [6]. Further, long-term studies have suggested that genetic alterations, environmental triggers, several viruses, and endogenous hormones may also play a role in ACC pathogenesis [6]. Pathological diagnosis stands on the identification of acinic cell differentiation (polyedric cells with abundant basophilic cytoplasm containing diastase-resistant PAS–positive zymogen granules), similar to the serous cellular elements found in the normal parotid gland [5, 7, 16]. These are epithelial pancytokeratin–positive cells characteristically immunoreactive for DOG1 antibody [16]. No molecular features are described as typical for ACC. High-grade transformation is a rare variant of ACC, anchoring an aggressive clinical behaviour and outcome [17]. Strong membrane beta-catenin and nuclear cyclin D1 immunoreactions have been associated with aggressive high-grade ACCs [16]. Empirical evidence has also shown that poor prognosis relies on blood vessel involvement, invasion to adjacent tissue, undifferentiated histological pattern, facial nerve involvement, and lymph node metastasis [18]. Surgical resection is the mainstay treatment; however, adjuvant radiotherapy may be advised for recurrent and undifferentiated tumours, with positive margins and locoregional dissemination [6]. Radiation monotherapy has also demonstrated effective results, mostly for inoperable tumours [6]. In addition, chemotherapy may be a useful therapeutic tool in cases of advanced ACC [7]. Nevertheless, current literature regarding the chemotherapy regimen of choice for parotid ACC is scarce, given the few cases depicted worldwide [7].

Our case remarkably reports a distinct presentation of parotid ACC, revealed by an ECS. The initial clinical hallmarks were classic of CS from ectopic ACTH-producing tumours, since this patient featured wasting, weakness, asthenia, peripheral oedema, hypertension, severe hypokalemia, and glucose intolerance. Moreover, the rapid onset and progression of symptoms along with the severity of endogenous hypercortisolism has increased the suspicion index, required to an early ECS diagnosis. Dynamic biochemical tests subsequently performed were concordant and confirmed the diagnosis. At this regard, one should bear in mind that ECS frequently represents an arduous diagnostic challenge as none of the biochemical parameters achieves 100% accuracy, and bilateral inferior petrosal sinus sampling (BIPSS) is often necessary, being considered the most reliable tool [3, 19].

Since the election treatment for ECS stands in complete resection of the tumour, localizing the source of ACTH production is crucial and remains a defiant endeavor for the majority of patients, even so for about 12% the source may not be identified [10]. As no single imaging modality has an ideal accuracy, it is recommended to add more than one imaging technique such as conventional CT, MRI, somatostatin receptor scintigraphy, or PET scan [19]. In this patient, a cervico-thoraco-abdominal CT scan revealed the parotid tumour, further depicted by MRI, also confirming liver metastasis. Additionally, octreoscan showed increased uptake in the parotid gland and mild expression in hepatic metastasis, albeit no uptake was observed in ^68^Ga-PET.

The histological and immunohistochemical examination of surgical specimen revealed an ACC with negative immunostaining for ACTH. At this respect, negative ACTH staining in malignant ectopic Cushing's tumours is thought to be related with a more aggressive pattern and worse prognosis [10, 20]. In effect, tumours owning a high ACTH secretory rate, with concomitant secretion of precursor fragments, can become depleted of its intracellular ACTH leading to a negative immunostaining, even though these patients exhibit high ACTH circulating levels, which may have been the case of our patient [20]. Moreover, herein a marked hypercortisolism was observed adding to the progressive clinical deterioration and adverse outcome. Accordingly, several lines of evidence point out that hypercortisolemia level is also strictly correlated with prognosis of ACTH secreting tumour [1]. Given the severity of hypercortisolism, the patient was kept under metyrapone, ketoconazole, and lanreotide. Actually, medical therapy is often warranted in order to control ECS, mainly until surgery. Current evidence indicates that adrenal steroidogenesis inhibitors as ketoconazole or metyrapone are preferred for their efficacy and safety. Somatostatin analogs have also been used to treat ECS, with controverse results [1]. Meanwhile, bilateral adrenalectomy was not performed in our patient since his clinical condition worsened, increasing the surgical risk. A rapid disease progression was documented despite the instituted chemotherapy, with a downhill clinical course.

In conclusion, the present case highlights an ECS unveiling a metastatic parotid ACC, with an aggressive and defiant behaviour. Noteworthy, contrary to the 5 previous cases published in the literature so far, this report represents the first in whose diagnosis of ECS came prior to ACC. Actually, since there are only few related cases depicted worldwide, current available literature is scarce and its pathophysiology has yet to be fully disclosed, standing this condition as a huge diagnostic and therapeutic challenge. Therefore, sharing these very uncommon cases may broad the knowledge to provide, in the future, a more comprehensive patient care.

## Figures and Tables

**Table 1:** Hormonal profile.

Hormonal study		Initial Assessment	After 1-month follow-up	After 1-month tumour ressection	Reference values
*Morning*	ACTH	206.8	422.3	200.7	<63.3 ng/L
	Cortisol	30.1	54.7	4.4	6.2–19.4 *μ*g/dL
*Midnight*	Serum cortisol	36.2	36.7	—	<7.5 *μ*g/dL
	Salivary cortisol	4.15	13.4	<0.018	<0.32 *μ*g/dL
	24h-UFC	6210.0	11068.2	14.9	36–137 *μ*g/24h
	1 mg overnight dexamethasone suppression test	42.6	—	—	<1.8 ug/dL

ACTH: adrenocorticotropic hormone; 24h-UFC: 24h-urinary free cortisol.

**Table 2:** Low- and high-dose dexamethasone suppression tests.

	Low-dose dexamethasone test			High-dose dexamethasone test		End	Reference Values
	Baseline	Day 1	Day 2	Day 3	Day 4	Day 5	
Cortisol 8 a.m.	38.3	44.7	44.6	36.9	32.2	32.0	6.2–19.4 *μ*g/dL
Cortisol 16 p.m.	38.1	47.5	38.2	36.5	28.7	—		
ACTH 8 a.m.	209.2	268.3	247.9	228.1	197.3	215.9	<63.3 ng/L
ACTH 16 p.m.	198.8	242.1	181.2	201.7	250.9	—		
24-h UFC	6900.3	3425.4	4148.0	3898.0	3097.7	—	36.0–137.0 *μ*g/24h

ACTH: adrenocorticotropic hormone; 24 h-UFC: 24 h-urinary free cortisol.

**Table 3:** Brief summary of the five cases previously published in the literature featuring ectopic ACTH secretion from metastatic ACC of the parotid gland.

	Case 1	Case 2	Case 3	Case 4	Case 5
	[11]	[12]	[13]	[14]	[10]
Age (years)	44	62	60	52	60
Sex (M/F)	M	F	M	F	F
Clinical features at presentation	Nervousness, insomnia, night sweats, nocturia, oedema, wasting, weakness, flushed and puffy face, cutaneous hyperpigmentation	Oedema, progressing fatigue, central obesity, mild hypertension	Acute confusional state, paranoid behaviour, hallucinations, oedema	Weakness, wasting, moonlike facies, buffalo hump, supraclavicular fat pads, thin skin, livedo reticularis, hypertension	Fatigue, oedema, weakness, central obesity, plethoric face, thin skin, purplish abdominal striae, exacerbated hypertension
Initial laboratory test	Exacerbated hyperglycemia, hypokalemia and metabolic alkalosis	Glucose intolerance, severe hypokalemia and metabolic alkalosis	Hypokalemia and metabolic alkalosis	Hypokalemia and metabolic alkalosis	Hypokalemia
Serum cortisol (µg/dL)	118 (RV: 12–18) (*morning*)	49.2 (RV: NA) (*morning*)	57.1 (RV:6.2–19.4) (*morning*)	171.6 (RV: <1.8) (*midnight*)	—
Morning serum ACTH (ng/L)	547 (RV: <120)	153 (RV: NA)	106 (RV: <40)	810 (RV: 10–50)	106 (RV: 7–63)
24h-UFC (µg/24h)	10700 (RV: 20–90)	—	5572.5 (RV: 7.2–32.6)	7720.4 (RV: <54.4)	1624.2 (RV: <52.9)
1 mg overnight dexamethasone suppression test (µg/dL)	97.0	***—***	***—***	***—***	28.5
Low- and high-dose dexamethasone suppression tests	***—***	***—***	Low-dose: positive	***—***	Low-dose: positive
Moment of ECS diagnosis	12 months after ACC diagnosis	7 months after ACC diagnosis	2 months after ACC diagnosis	6 months after ACC diagnosis	36 months after ACC diagnosis
Treatment	(i) Total parotidectomy combined with left radical neck dissection; radiotherapy 9 months later for local recurrence	(i) Right total parotidectomy with sacrifice of the facial nerve and right facial sling; postoperative radiotherapy	(i) Left parotidectomy and radical neck dissection; postoperative radiotherapy; palliative chemotherapy with gemcitabine and docetaxel	(i) Total parotidectomy with right neck dissection; postoperative radiotherapy; palliative chemotherapy with single-agent doxorubicin	(i) Surgical resection (information about neck dissection or adjuvant therapy NA)
	(ii) No steroidogenesis inhibitors employed	(ii) No steroidogenesis inhibitors employed	(ii) Metyrapone	(ii) Ketoconazole, somatostatin analogues	(ii) Metyrapone
Clinical course	Rapid clinical deterioration	Rapid clinical deterioration	Clinical and biochemical improvement after 2 cycles of chemotherapy	Rapid clinical deterioration	Rapid clinical deterioration
Time from ECS diagnosis to disease-associated death (weeks)	6	4	NA	3	NA

ACTH: adrenocorticotropic hormone; ACC: acinic cell carcinoma; M/F: male/female; RV: reference value; 24h-UFC: 24h-urinary free cortisol; ECS: ectopic Cushing syndrome; NA: not available.
